# Inflammation-Induced Tumorigenesis and Metastasis

**DOI:** 10.3390/ijms22115421

**Published:** 2021-05-21

**Authors:** Sana Hibino, Tetsuro Kawazoe, Hidenori Kasahara, Shinji Itoh, Takatsugu Ishimoto, Mamiko Sakata-Yanagimoto, Koji Taniguchi

**Affiliations:** 1 Research Center for Advanced Science and Technology, Department of Inflammology, The University of Tokyo, Tokyo 153-0041, Japan; sana.hibino@inflammology.rcast.u-tokyo.ac.jp; 2 Department of Microbiology and Immunology, Keio University School of Medicine, Tokyo 160-8582, Japan; kzoe922@gmail.com; 3 Department of Surgery and Science, Graduate School of Medical Sciences, Kyushu University, Fukuoka 812-8582, Japan; itoh.shinji.453@m.kyushu-u.ac.jp; 4 National Center for Global Health and Medicine, Department of Stem Cell Biology, Research Institute, Tokyo 162-8655, Japan; kasahara.hideno@gmail.com; 5 Division of Hematology, Department of Medicine, Keio University School of Medicine, Tokyo 160-8582, Japan; 6 Department of Pathology, New York University School of Medicine, New York, NY 10016, USA; 7 Gastrointestinal Cancer Biology, International Research Center of Medical Sciences (IRCMS), Kumamoto University, Kumamoto 860-0811, Japan; taka1516@kumamoto-u.ac.jp; 8 Department of Hematology, Faculty of Medicine, University of Tsukuba, Tsukuba 305-8575, Japan; sakatama@md.tsukuba.ac.jp; 9 Department of Pathology, Faculty of Medicine and Graduate School of Medicine, Hokkaido University, Sapporo 060-8638, Japan

**Keywords:** inflammation, cancer, metastasis, tumor microenvironment, immunosuppression

## Abstract

Inflammation, especially chronic inflammation, plays a pivotal role in tumorigenesis and metastasis through various mechanisms and is now recognized as a hallmark of cancer and an attractive therapeutic target in cancer. In this review, we discuss recent advances in molecular mechanisms of how inflammation promotes tumorigenesis and metastasis and suppresses anti-tumor immunity in various types of solid tumors, including esophageal, gastric, colorectal, liver, and pancreatic cancer as well as hematopoietic malignancies.

## 1. Introduction

Inflammation is the body’s immunological defense mechanism against harmful irritants, from the elimination of injury factors to regeneration of injured tissue [1]. Sustained inflammatory stimuli and immune cell activation lead to chronic inflammation, which involves repeated tissue destruction and regeneration for a long period of time. It has been argued since the 19th century that there are similarities and connections between cancer and chronic inflammation. In 1863, the German pathologist Rudolf Virchow first documented immune cell infiltration within neoplastic tissue and suggested that chronic inflammatory responses contribute to tumor development [2]. In 1915, the Japanese pathologist Katsusaburo Yamagiwa, inspired by Virchow’s findings, demonstrated that artificially induced chronic inflammation could lead to tumor formation in an animal model [3]. During the 1900s, many scientists published papers supporting Virchow’s theory. For example, the idea that non-specific inhibition of inflammation by non-steroidal anti-inflammatory drugs might inhibit cancer occurrence or growth was suggested in the 1970s [4]. Harold F. Dvorak described tumors as “Wounds that do not heal”, implying that biological processes involved in tumor development are similar to those in wound healing and tissue regeneration after injury [5,6]. In the past 20 years, inflammation has been broadly accepted as a hallmark and cause of cancer [7]. Owing to advances in molecular biology and the development of genetically modified mice, the molecular and cellular landscape of inflammation during tumorigenesis, including detailed function of various types of immune cells and networks of complex signaling pathways orchestrated by multiple cytokines, chemokines, and growth factors, was uncovered [8]. Additionally, developments in comprehensive analyses, represented by next-generation sequencing (NGS) and single cell technologies, further unraveled unknown inflammatory aspects of cancer [9,10,11].

Inflammation is a critical contributing factor in the pathology of most solid and hematopoietic malignancies. Therefore, understanding molecular mechanisms connecting inflammation to tumorigenesis and metastasis is essential for discussing the development of anti-cancer therapeutic strategies. This review outlines mechanisms of cancer-related inflammation, including the latest findings in this field, and discusses the role of inflammation for individual cancer types.

## 2. Triggers of Inflammation during Tumorigenesis

Only 10% of cancer cases are attributable to germline mutations; most cancers are caused by acquired factors, such as environmental cues, which are often closely associated with chronic inflammation [8]. Importantly, in addition to tumor-extrinsic factors, tumor-intrinsic factors can be the origins of the tumor-promoting inflammatory loop [12]. We will discuss potential triggers for the induction of inflammation during tumorigenesis.

### 2.1. Inflammation Caused by Chronic Infection and Autoimmunity

Approximately 20% of cancer cases are accompanied with persistent infection [8], such as *Helicobacter pylori*-induced gastritis and hepatitis B/C virus (HBV or HCV)-induced hepatitis, which increase the risk of gastric cancer (GC) and hepatocellular carcinoma (HCC), respectively [13]. Pathogens, such as viruses and bacteria, are rapidly eliminated by the host immune system in principle; however, tumorigenic pathogens often evade host defense mechanisms and establish persistent infection, leading to tumor-promoting chronic inflammation.

Chronic inflammation induced by autoimmune disorders also enhances the risk of tumorigenesis. For example, patients with inflammatory bowel disease (IBD) are at a higher risk of colorectal cancer (CRC), due to the pro-neoplastic effects of chronic intestinal inflammation [14]. Two Japanese research groups have shown that the inflamed epithelium of patients with ulcerative colitis undergoes selective expansion of cell clones with mutations in interleukin (IL)-17A signaling-related genes, which are resistant to inflammatory damage [15,16]. However, these somatic mutations are not found in CRC tissues, suggesting negative selection.

Notably, not all chronic inflammatory diseases are linked to cancer development; the site of the tissue or organ at which inflammatory responses occur is critical—for example, joint or muscle inflammation rarely leads to cancer development [8].

### 2.2. Inflammation Caused by Environmental and Lifestyle Factors

Humans are exposed to various environmental factors, which can trigger chronic inflammation, albeit at a low intensity in many cases [8]. For example, inhalation of particulate material from tobacco smoke, asbestos, and silica promotes lung cancer and mesothelioma by causing airway and lung inflammation [17,18]. Lifestyle factors, such as poor eating habits, can also induce tumor-promoting chronic inflammation, as evidenced by the increase in risk of various cancer types in cases with obesity and excessive lipid accumulation [19,20]. Mounting evidence suggests that gut microbiota dysbiosis and gut barrier dysfunction induced by obesity and a high-fat diet (HFD) trigger low-grade persistent inflammation, leading to gastrointestinal cancers [21]. According to a report, excessive intake of fructose, a widely-used sweetener, causes intestinal barrier deterioration, and the leakage of the gut microbiota-derived endotoxin into the circulation, which acts as an inflammatory trigger for hepatosteatosis and nonalcoholic steatohepatitis (NASH), thereby predisposing the patient to HCC [22,23]. Considering the spread of a Western-style diet globally, carcinogenesis due to dietary and nutritional disorders will become an increasingly serious problem.

### 2.3. Aging-Associated Inflammation

Aging has recently been recognized as a chronic and systemic low-grade inflammatory process (inflammaging), accounting for higher cancer morbidity in elderly people [24,25]. Multiple factors, including long-held lifestyles, immunosenescence, and cellular senescence, are implicated in age-related inflammation and subsequently, cancer development. For example, senescent cells, which accumulate as a result of aging, secrete tumor-promoting inflammatory proteins, the so-called senescence-associated secretory phenotype (SASP) factors, which include IL-6 and IL-8 [26,27]. A recent report suggested the use of chimeric antigen receptor (CAR) T cells, which target senescent cells, could be an effective cancer treatment strategy [28].

Interestingly, large-scale genomic analysis revealed age-related expansion of cell clones with mutations in cancer driver genes in physiologically normal esophageal epithelia [29]. According to this report, heavy smoking and drinking accelerate the age-related remodeling of esophageal epithelia, suggesting the contribution of inflammaging to esophageal cancer.

### 2.4. Cancer-Elicited Inflammation

In contrast to other inflammation types, which occur prior to tumor formation, “cancer-elicited inflammation (CEI)” is provoked after tumor initiation [12]. For example, only 2% of CRCs are preceded by apparent intestinal inflammation, such as IBD; however, prominent immune cell infiltration and enhanced expression of inflammatory cytokines and chemokines are observed in the majority of sporadic CRC tissues [14]. Various tumor-intrinsic factors act as triggers of CEI. In addition to cancer cell-autonomous effects, activation of oncogenes, such as *KRAS* and *MYC*, and inactivation of tumor suppressors, such as *TP53*, induce transcriptional programs that lead to the establishment of the tumor-promoting microenvironment through the excessive production of pro-inflammatory cytokines and chemokines, recruitment of immune cells, and induction of angiogenesis [30,31,32]. For example, loss of p53 in breast cancer cells induces the secretion of WNT ligands, which stimulate tumor-associated macrophages (TAMs) to produce IL-1β, resulting in metastatic progression-potentiating systemic neutrophilic inflammation [33].

Most solid tumors are exposed to severe microenvironments, such as hypoxia and nutrient starvation, due to abnormal angiogenesis, which leads to the massive necrotic cell death of tumor cells [34]. Necrotic tumor cells release their intracellular components, some of which act as functional inflammatory mediators, the so-called damage-associated molecular patterns (DAMPs), such as IL-1α, ATP, and high mobility group box 1 (HMGB1) [35]. DAMPs activate the innate immune response by interacting with pattern recognition receptors (PRRs), such as toll-like receptors (TLRs), and contribute to tumor progression. These types of CEI can be categorized into “sterile inflammation” induced in the absence of apparent pathogenic infection.

However, pathogens, such as bacteria, and bacterial products, also act as triggers of CEI. For example, loss of adenomatous polyposis coli (*APC*) gene in CRCs results in the deterioration of the intestinal barrier, and the subsequent microbial invasion into the tumor bed triggers inflammatory responses, thereby promoting tumor growth [36]. Thus, loss of tissue organization due to malignant transformation is a key driver of CEIs.

### 2.5. Inflammation Caused by Cancer Therapy

Although it seems contradictory, attempts to eradicate tumor cells induce tumor-promoting inflammatory responses in some situations. Chemotherapeutic agents and radiation cause massive necrotic cell death, and tumor cell debris or released DAMP molecules stimulate the production of pro-inflammatory cytokines from immune cells, which support tumor growth, angiogenesis, and metastasis [37,38]. This phenomenon probably resembles wound healing and tissue repair in normal tissues after injury. Anti-cancer therapy-induced inflammation also contributes to therapy resistance. For example, administration of 5-fluorouracil (5-FU) provokes tumor-promoting inflammatory responses, but the neutralization of cytokines, such as IL-17A, enhances treatment responsiveness [39]. Importantly, normal tissues, such as the intestines, are also damaged by chemotherapies, and bacterial translocation resulting from the disruption of intestinal barrier integrity may activate systemic inflammation, which accelerates tumor growth. Thus, cytotoxic cancer therapy is a double-edged sword.

By contrast, cancer cell death induced by anti-cancer therapy is often accompanied by an increased release of tumor antigens, which may enhance anti-tumor immune responses [8]. Thus, whether therapy-induced inflammation has positive effects on tumor progression is controversial; multiple factors, including types of cancer cells and anti-cancer agents used, may determine the outcome. Notably, the way cell death occurs is important; apoptosis and autophagic cell death are less inflammatory, whereas necrosis and necroptosis, which result in the release of DAMPs, are more potent inducers of inflammation [40,41].

## 3. Cell Types Engaged in Cancer-Associated Inflammation

The tumor microenvironment (TME) is composed of heterogeneous cell populations, including cancer cells, immune cells, and stromal cells, such as fibroblasts and endothelial cells. The dynamic crosstalk between diverse cell types, through direct cell–cell contact or soluble factors, such as cytokines, chemokines, and growth factors, creates an inflammatory niche to support cancer development. What is important is these cells are highly plastic; they continuously change their phenotypic and functional characteristics to support tumor development [42]. We summarize the features and roles of components of the TME.

### 3.1. Myeloid Cells

TAMs are one of the most frequently observed immune cell subsets in the TME and play indispensable roles in cancer-associated inflammation, especially as a source of inflammatory cytokines [43]. Characteristic features of this cell population include functional “polarization” regulated by environmental cues, namely M1 and M2 subtypes. M1-type macrophages have a pro-inflammatory phenotype with cytotoxic activities and are characterized by the production of inflammatory cytokines, such as IL-1, IL-6, IL-12, and tumor necrosis factor (TNF), reactive oxygen species (ROS), and nitric oxide (NO). By contrast, M2-type macrophages play critical roles in the resolution of inflammatory responses and are characterized by the expression of molecules associated with immunosuppression (IL-10, TGF-β, prostaglandin E_2_ (PGE_2_), and arginase-1); angiogenesis (IL-8, vascular endothelial growth factor A (VEGF-A), and matrix metalloprotease 9 (MMP-9)); and cell proliferation and tissue repair (insulin-like growth factor 1 (IGF-1) and amphiregulin) [44]. In the TME, tumor-derived factors, such as lactic acid, which is the end product of the Warburg effect, induce M2-like polarization of macrophages; most TAMs are believed to be classified into the M2 subtype, which participates in the promotion of tumor growth and angiogenesis [45]. However, “M1-cytokines” derived from TAMs are the main source of tumor-promoting inflammatory cytokines in the TME. The general definition of M1 and M2 macrophages is based on gene expression patterns, but in vivo TAMs can express both M1- and M2-related genes—the M1/M2 paradigm reaches its limits in a complex context, such as the TME, so a new perspective may be required for the functional classification of TAMs [42]. Interestingly, according to a recent report, pyrimidine nucleotides released from tumor-educated macrophages confer resistance of pancreatic cancer cells to gemcitabine, unveiling a new role for TAMs [46].

Tumor-associated neutrophils (TANs), which are central players in acute inflammation, also engage in cancer-associated chronic inflammation. High systemic neutrophil-to-lymphocyte ratios (NLRs) are associated with poor overall survival in most human epithelial tumor types [47], and systemic neutrophilic inflammation in patients with cancer supports cancer cell metastasis [48,49]. Interestingly, studies have revealed that neutrophils accumulating in tumor-bearing animals and humans are “pathologically activated,” and can promote tumor growth by suppressing effective anti-tumor immune responses and contributing to the formation of premetastatic or metastatic niches [49,50]. These neutrophils are recognized as polymorphonuclear myeloid-derived suppressor cells (PMN-MDSCs); elevated accumulation of MDSCs in patients with cancer could act as an indicator of poor outcome and unresponsiveness to cancer therapies [51]. Interestingly, recent reports demonstrated that the endoplasmic reticulum (ER) stress response and cellular lipid accumulation regulate the functional reprogramming of neutrophils in the TME [52,53]. In addition to PMN-MDSCs, another subtype of MDSCs, which is morphologically similar to monocytes (monocytic MDSCs; M-MDSCs), was identified in tumor-bearing hosts. Kwak et al. reported that M-MDSCs could differentiate into tumor-promoting macrophages, which are characterized by the expression of S100A9 [54].

Dendritic cells (DCs) constitute a rare immune cell population within the TME, but have been widely recognized as the most potent antigen-presenting cells that are capable of inducing tumor-specific T cell responses. However, such anti-tumor functions are often compromised in the context of the TME, and they are polarized into tumor-promoting phenotypes [55].

### 3.2. Lymphocytes

T lymphocytes are an immune cell population that frequently infiltrate the TME. T lymphocytes are classified into two major groups based on the expression of T cell receptors, namely αβ and γδ T cells. Most T cells are αβ T cells, which are further classified into CD8^+^ cytotoxic T and CD4^+^ helper T (Th) cells, which include Th1, Th2, Th9, Th17, Th22, Tfh, and regulatory T (Treg) cells [56]. CD8^+^ T and Th1 cells are central players in anti-tumor immunity, which can destroy tumor cells. By contrast, Th17 and Th22 cells serve as a critical source of pro-tumorigenic cytokines IL-17 and IL-22, respectively, during intestinal tumorigenesis [39,57]. Treg cells are an immunosuppressive T cell subset that suppress effective anti-tumor responses and promote tumor progression [58]; however, Tregs can be converted into a tumor-promoting inflammatory phenotype similar to Th17 and Th22 under specific pathological conditions [59,60]. CD4^+^ T cells have recently gained attention as an important cell population in inflammaging [61,62], suggesting their contribution to age-dependent tumorigenesis.

Innate lymphoid cells (ILCs), a novel population of innate lymphocytes, regulate tissue homeostasis, inflammation, tumorigenesis, and tumor surveillance [63,64,65,66]. ILCs are divided into three groups: group 1 ILCs, which include natural killer (NK) cells and ILC1s, group 2 ILCs (ILC2s), and group 3 ILCs, which include lymphoid tissue-inducer (LTi) cells and ILC3s. Group 1 ILCs are predominantly anti-tumorigenic, whereas group 2 and 3 ILCs are mostly considered to be pro-tumorigenic [63]. Although the role of NK cells in cancer is well established, the studies on other ILC subsets in cancer are limited and their roles in inflammation-induced tumorigenesis and metastasis as well as anti-cancer immunity are now under investigation.

### 3.3. Tumor Endothelial Cells

Tumor endothelial cells (TECs) refer to cells that line tumor blood vessels, which support tumor development by providing nutrients and oxygen and acting as conduits for cancer cell dissemination [67]. Angiogenesis involves the development of new blood vessels, and sustained angiogenesis is a hallmark of cancer [7]. Hypoxia is the major trigger of tumor angiogenesis and hypoxia-inducible factor 1 (HIF-1) acts as a key regulator of this process [68]. Importantly, TAMs play a critical role in tumor angiogenesis by producing pro-angiogenic factors, such as VEGF-A, in response to hypoxic signals [69]. Interestingly, the interaction between tumor cells and TECs through inflammatory mediators could regulate the intravasation and extravasation of metastatic cancer cells [70,71].

### 3.4. Cancer-Associated Fibroblasts

Cancer-associated fibroblasts (CAFs) are the most prominent stromal cell type in the microenvironment of various solid cancers and are induced during the transition from normal fibroblasts through reciprocal tumor stroma signaling. CAFs have been shown to facilitate tumor growth by several mechanisms: (1) direct stimulation of cancer cell proliferation through the secretion of growth factors, (2) promotion of tumor cell invasion by inducing angiogenesis and remodeling the extracellular matrix (ECM), and (3) induction of tumor-promoting inflammation through the secretion of cytokines and chemokines, which mediate the recruitment and functional regulation of immune cells within the TME [72]. For example, the activation of the NLRP3 inflammasome in CAFs and the subsequent IL-1β secretion promotes progression and lung metastasis of breast cancer [73]. In contrast, the pro-inflammatory cytokines TNF, IL-1α, and IL-1β induce cellular senescence in the CAFs. Subsequently, the expression of IL-6 is maintained at a high level through the demethylation of H3K27me3 mediated by EZH2 downregulation in senescent CAFs, enhancing peritoneal dissemination of GC [74]. The simultaneous high levels of these pro-inflammatory cytokines are significantly associated with a poor prognosis of patients with GC [75]. Studies have reported that CAFs are a heterogeneous cell population consisting of different cell subsets with different functions [76,77,78]. According to a report from Öhlund et al. [79], there are two spatially and functionally distinct CAF populations, namely inflammatory CAFs (iCAFs) and myofibroblastic CAFs (myCAFs). iCAFs are characterized by the low expression of α-SMA and high expression of inflammatory mediators, such as IL-6, IL-11, and leukemia inhibitory factor (LIF); myCAFs exhibit contrasting properties. Therefore, specifically targeting pro-tumorigenic subpopulations of CAFs could be a promising therapeutic strategy for various solid cancers.

## 4. Multifaceted Role of Inflammation during Tumorigenesis

**Figure 1:**
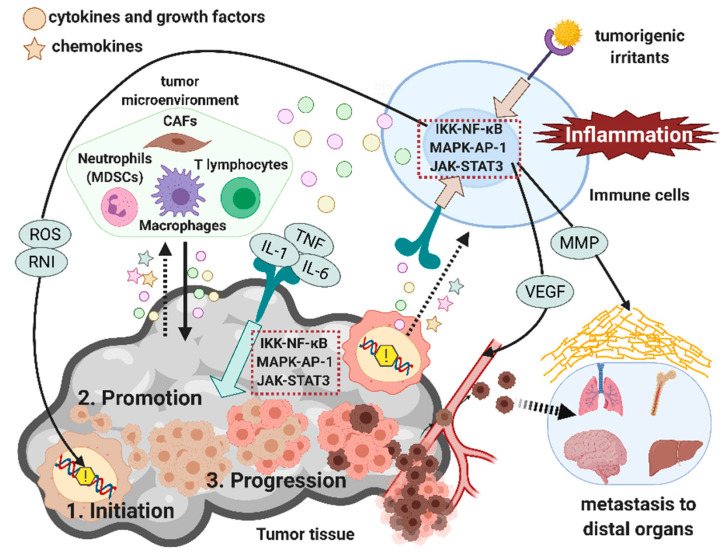
Chronic inflammation promotes tumor progression.

Chronic inflammation impacts cancer development—from tumor initiation and promotion to progression and metastasis. Here, we outline the multifaceted role of inflammation at each stage of tumor development (Figure 1).

Persistent exposure to tumorigenic irritants, such as infected virus and gut microbes, often activates inflammatory signaling, leading to tumor-promoting chronic inflammation.

Inflammatory cells often act as a critical source of various tumor-promoting inflammatory mediators, including ROS, cytokines such as TNF, IL-1, and IL-6, and growth factors that directly or indirectly support the entire process of tumor development. Tumor cells also help amplify this inflammatory loop. For example, the dysregulation in cancer-associated genes results in the excessive production of chemokines and cytokines, further recruiting and activating various immune cells (dashed arrows).

### 4.1. Role of Inflammation in Tumor Initiation

Tumor initiation requires accumulation of genetic mutations, epigenetic alterations, or both in cancer-related genes in normal cells. The inflammatory microenvironment largely contributes to this process. Activated inflammatory cells, such as macrophages and neutrophils, are major sources of ROS and reactive nitrogen intermediates (RNIs), which induce DNA damage and genomic instability. Canli et al. demonstrated that myeloid cell-derived ROS triggers genome-wide mutations in epithelial cells and stimulates their malignant transformation [80]. Cytokines released from inflammatory cells also contribute to mutagenesis by elevating intracellular ROS and RNI in premalignant cells. Such inflammation-induced mutagenesis is reported for several important cancer-related genes, such as mismatch repair (MMR) response genes and tumor suppressor *TP53* [8]. Chronic inflammation can also induce chromosomal instability and dysregulation of the epigenetic machinery, such as DNA- and histone-modifying enzymes, microRNA, and long non-coding RNA (lncRNA), leading to tumor development.

Conversely, inflammation can be triggered by genomic DNA damage. For example, carcinogen-induced genotoxic stress evokes the innate immune DNA-sensing pathway, leading to inflammation-induced skin carcinogenesis [81,82]. Wilson et al. found that the gut bacterial genotoxin colibactin alkylates DNA *in vivo*, and colibactin-mediated DNA damage might contribute to CRC development [83].

Moreover, chronic inflammation promotes tumor initiation by conferring stem cell-like characteristics on premalignant cells and forming niches that support the survival and maintenance of tumor-initiating cells, thereby expanding targets for mutagenesis [8].

### 4.2. Role of Inflammation in Tumor Promotion

During tumor promotion, malignant-transformed cells undergo aberrant proliferation to form the primary tumor. At this stage, inflammatory cells act as a critical source of cytokines and growth factors to promote tumor cell survival and proliferation. Myeloid cell-specific inactivation of NF-κB signaling, a central axis of pro-inflammatory cytokine production, leads to tumor growth suppression in multiple cancer models [84,85]. The experiments using mice with knocked-out genes encoding various cytokines or cytokine receptors also revealed the contribution of these factors to tumor promotion [86]. Inflammatory signals impact not only tumor cells but also the components of tumor stroma, including immune cells, fibroblasts, and endothelial cells, and shape the TME, which supports further tumor progression [8].

### 4.3. Role of Inflammation in Tumor Progression and Metastasis

During tumor progression, tumor cells acquire malignant characteristics, such as metastatic ability, resulting in uncontrollable, and life-threatening states. One of the most important events in this phase is epithelial–mesenchymal transition (EMT), through which epithelial cancer cells acquire mesenchymal characteristics with enhanced cell motility and migratory activities [87,88]. EMT is observed not only during wound healing and tissue fibrosis after tissue injury but also during cancer progression and metastasis. Multiple inflammatory mediators, such as TNF, IL-1β, IL-6, IL-11, and IL-8, are reportedly potent inducers of EMT [89].

Remodeling of the tumor stroma is necessary for the effective migration and invasion of cancer cells, and inflammation also participates in this process. For example, TAMs promote cancer cell metastasis by producing MMPs, which destroy cell–cell adhesions and the extracellular matrix [90]. Moreover, tumor cell migration and metastasis to specific distal organs is guided by chemokine gradients formed within the TME and sensed by chemokine receptors, in a manner similar to leukocyte trafficking [91]. Several pro-inflammatory cytokines, such as TNF and IL-1β, which are enriched in the TME, induce the expression of chemokines, such as CXCL1 (keratinocyte-derived chemokines), CXCL5, CXCL8 (IL-8), CCL2 (MCP-1), and CCL5 (RANTES) [92,93].

Cancer cell metastasis occurs through lymphatic and blood vessels [8]. Activated macrophages are a source of angiogenesis- and lymphangiogenesis-stimulating factors, such as members of the VEGF family. Once metastatic cancer cells enter the circulation, inflammatory mediators released by immune cells support their survival and colonization in the target organ. Albrengues et al. demonstrated that sustained experimental lung inflammation induced by exposure to tobacco smoke or lipopolysaccharide (LPS), and the accompanying formation of neutrophil extracellular traps (NETs), converted dormant cancer cells to aggressively growing metastases in mice [94].

## 5. Tumor-Promoting Inflammatory Signaling

Cytokines, chemokines, and growth factors play dominant roles in the formation of the inflammatory milieu within the TME and promote tumor progression by directly acting on tumor cells or by indirectly modulating other components of the TME. The production and function of a diverse array of inflammatory mediators involves complex signaling networks orchestrated by multiple signaling molecules and transcription factors. Of these, the IKK-nuclear factor-κB (NF-κB), Janus kinase (JAK)-signal transducer and activator of transcription 3 (STAT3), and mitogen-activated protein kinase (MAPK)-activator protein-1 (AP-1) axes play dominant roles [8,95,96].

NF-κB belongs to a family of dimeric DNA-binding transcription factors; their activities are tightly controlled by inhibitor of nuclear factor-kB kinase subunit β (IKKβ) [95]. The IKK–NF-κB axis regulates the expression of various types of gene sets associated with cancer and inflammation, including pro-inflammatory cytokines (*TNF, IL1B*, and *IL6)*; growth factors (*CSF2* (GM-CSF), *SPP1* (osteopontin), and *VEGFA*); chemokines (*CXCL8* (IL-8), *CXCL1, CXCL2*, *CCL2* (MCP-1), and *CCL5* (RANTES)); MMPs; pro-inflammatory enzymes (*PTGS2* (cyclooxygenase-2 (COX-2)and *NOS2* (inducible NO synthase (iNOS)); cell adhesion molecule (*vascular cell adhesion molecule 1* (*VCAM1*)); proto-oncogenes (*MYC* (c-Myc) and *CCND1* (cyclin-D1)); and anti-apoptotic genes (*BCL2L1* (Bcl-_X_L) and *BCL2* (Bcl-*2*)) [95]. Notably, constitutive activation of the IKK–NF-κB axis has been observed in many types of cancers, both in cancer cells and surrounding components. Receptors activating NF-κB through their downstream signal transduction pathway include PRRs, such as TLRs and Nod-like receptors. Various tumorigenic irritants, such as pathogens, gut microbes, and DAMPs, act as persistent stimuli for PRRs and turn on the NF-κB-dependent gene expression program. In addition, pro-inflammatory cytokines, such as TNF and IL-1, can induce NF-κB activation through their cognate receptors and create a positive feedback loop [95].

STAT3 was first described as a DNA-binding activity in IL-6-stimulated hepatocytes [96]. Hyperactivation of STAT3 in both cancer cells and non-cancerous cells within the TME is observed in most human malignancies; its association with poor patient outcome has also been reported [97,98]. Activation of STAT3 is mainly triggered by IL-6 family cytokines (IL-6, IL-11, LIF, and oncostatin M), IL-22, IL-23, and various growth factors (epidermal growth factor (EGF) and platelet-derived growth factor (PDGF)) through their cognate receptors; the subsequent phosphorylation by JAK enables binding of STAT3 to the target sequence to initiate transcription [97,99]. STAT3 induces various gene sets associated with tumor progression, many of which are common targets of NF-κB [95].

AP-1 is a dimeric DNA-binding transcription factor, comprising Jun and Fos, and acts downstream of MAPK signaling. Similar to NF-κB, the MAPK–AP-1 axis is activated downstream of receptors for inflammatory cytokines (TNF and IL-1) and PRRs, and induces the expression of a series of target genes.

The individual actions of NF-κB, STAT3, and AP-1 are insufficient to govern the gene expression programs that sustain tumor-promoting inflammation; crosstalk and cooperative action is important, and interaction with many other transcription factors and associated signaling cascades (e.g., p53, HIF-1α, and WNT-β-catenin) has also been reported [95,100]. Interestingly, in gastrointestinal tumors, IL-6 family cytokines also activate YAP and downstream Notch signaling via their common receptor glycoprotein 130 (gp130)/IL6ST, in addition to the JAK–STAT3 pathway. YAP and Notch are transcriptional regulators that control tissue growth and regeneration; thus, cancer-associated inflammation and tissue regeneration are linked through gp130 signaling [101,102,103].

## 6. Role of Inflammation in Cancer Types

### 6.1. Esophageal Cancer

Esophageal squamous cell carcinoma (ESCC) and esophageal adenocarcinoma (EAC) are the two main histological subtypes of esophageal cancer, which account for more than 95% of all the esophageal cancer cases. ESCC is the most common subtype of esophageal cancer worldwide, especially in Asia and parts of Africa, and EAC is the predominant type of esophageal cancer in Western countries [104].

The major risk factors for ESCC include tobacco and alcohol consumption, both being closely associated with inflammation, but other environmental factors also play a role in the development of ESCC, such as the consumption of hot beverages, nutritional deficiencies, and limited intake of fruits and vegetables [105]. People who consume both tobacco and alcohol are especially at risk. The reason is unclear but some explanations for this synergy are as follows. Acetaldehyde, which is the first metabolite of ethanol and a constituent of tobacco smoke, is a known local carcinogen, and the addition of active smoking during ethanol consumption increases levels of acetaldehyde compared to ethanol consumption alone [106]. Esophageal intraepithelial neoplasia (IEN), a precancerous lesion of ESCC, acts as a site of chronic inflammation, with a significantly increased risk of developing into ESCC [107]. Esophageal IEN tissues and ESCCs have similar mutations and markers of genomic instability, including apolipoprotein B messenger RNA editing enzyme, catalytic polypeptide-like (APOBEC), which causes DNA damage-related mutagenesis [108]. High expression of γH2AX is also detected in esophageal IEN, which confirms DNA damage as an important mutation pattern of esophageal carcinogenesis. Defects in DNA damage repair and cell cycle regulation exist both in the early stages and during ESCC progression; the defects could help damaged cells escape from early barriers of tumorigenesis and could underlie the initial tumor clone formation [109]. *TP53* and *CDKN2A* were identified as the early mutated driver genes in esophageal IEN; amplifications of *CCND1* and *SOX2* and mutations in *NOTCH1* are common mutations in ESCC [105,109]. ESCC cells can secrete IL-6 and other pro-inflammatory cytokines, including IL-8 and LIF, which have been implicated in cancer progression and immune evasion through the activation of STAT3 and YAP [103,110].

The primary risk factor for EAC is gastroesophageal reflux disease (GERD). GERD is a major cause of reflux esophagitis, which occurs when gastric acid, bile acids, and other harmful substances in gastric juice flow backward into the esophagus [111]. Barrett’s epithelium, a metaplasia from squamous epithelium into columnar epithelium, originates from GERD, and can develop to EAC and esophageal neuroendocrine carcinoma [112]. Activation of NF-κB, which is reportedly overexpressed after exposure to bile acids, has been implicated in carcinogenesis from Barrett’s esophagus [113]. This carcinogenic process is likely initiated by the production of N-nitroso-bile acid, which is mutagenic and promoted by sustained chronic inflammation [114]. Barrett’s esophagus is thought to progress to EAC in stages, with loss of *CDKN2A* followed by *TP53* inactivation and aneuploidy [115].

Tumor mutation burden in esophageal cancer is relatively high among gastrointestinal cancers, and the response to immune checkpoint inhibitors (ICIs) was estimated to be high [116]. In fact, clinical trials seem to support the strong preclinical rationale of a potential role of immunotherapy for esophageal cancer in both advanced and localized disease [117].

### 6.2. Gastric Cancer

Infections with *H. pylori*, a Gram-negative helical bacterium, have been identified as a strong risk factor for GC and other gastroduodenal disorders, such as chronic gastritis, gastric ulcer, and duodenal ulcer, and *H. pylori* is classified as a class 1 carcinogen by the International Agency for Research on Cancer [118]. *H. pylori* is unique because it survives in the highly acidic environment of the human stomach, where the pH drops to as low as 1.8 during digestion [119]. Under highly acidic conditions, *H. pylori* maintains survivability by releasing urease and urease cofactors, namely NixA and NikR, to produce NH_4_^+^, which neutralize the acidity [120]. Patients infected with *H. pylori* develop acute or chronic gastritis, which may progress to gastric ulcer, intestinal metaplasia, and gastric carcinoma [121]. *H. pylori* infection increases NF-κB activity and the nuclear translocation of NF-κB p50/p65 heterodimers to induce the activation of the NF-κB pathway [122]. The expression of pro-inflammatory cytokines, such as IL-8 and IL-17, is induced by the activated NF-κB pathway to initiate gastric neoplasia [122]. Moreover, the *H. pylori*-mediated activation of NF-κB and AP-1 regulates transcription of oncogenes, such as *CTNNB1* (β-catenin) and *MYC*, and mediates the hyperproliferation of gastric cells [123]. In addition to NF-κB activation, epigenetic alterations during chronic inflammation by *H. pylori* are also involved in gastric carcinogenesis [124,125]. This process is widely accepted as an epigenetic field defect in various types of cancer [126].

GCs have been classified according to several molecular features. The Cancer Genome Atlas research network performed the most comprehensive integrated genome-wide analysis to propose four molecularly distinct GC subtypes, namely Epstein–Barr virus-positive (EBV^+^), microsatellite instable (MSI), genomically stable, and chromosomal unstable (CSI) [127]. EBV is a gamma-herpesvirus, which infects B cells and mucosal epithelia and induces cellular proliferation [128]. EBV^+^ GCs are characterized by recurrent *PIK3CA* mutations, extreme DNA hypermethylation, and *JAK2* amplification [127]. The prognosis of EBV^+^ GC is better than that for EBV^−^ GC probably due to enhanced CD8^+^ T cell infiltration [129]. A study reported that both MHC-II genes and genes involved in antigen presentation are upregulated in EBV^+^ GCs compared to other GC subtypes [130]. Thus, EBV^+^ GC cells may functionally contribute to the highly immunogenic TME through interferon (IFN)-induced antigen presentation. Consistently, GC patients with EBV benefit from anti-PD-L1 immunotherapy and have better 5-year survival statistics [131].

CAFs produce several cytokines such as IL-6, IL-11, and IL-22 and create a favorable environment for cancer. IL-6 enhances cancer cell migration and EMT by activating STAT3 and IL-11 to facilitate resistance to chemotherapeutic drugs [132,133]. IL-22 promotes cancer cell migration and invasion via STAT3, ERK, JNK, and Akt pathways [134,135,136].

In addition to PD-L1 immunotherapy, PD-1 inhibition was found to increase survival and enhance the efficacy of ramucirumab, an anti-VEGFR-2 monoclonal antibody [137]. Although preclinical research on targeting the immune system through other markers, including CAR-T therapy, tumor-infiltrating lymphocytes (TIL) therapy, IFNs, and ILs, is being conducted, further work on GC subtypes to identify these vulnerable groups is warranted [138].

### 6.3. Colorectal Cancer

CRCs appear to develop through various premalignant stages, namely adenoma, familial adenomatous polyposis, Peutz–Jeghers syndrome, juvenile polyposis, and dysplasia in IBD [139]. The precursors of almost all sporadic CRCs are colorectal adenomas, which develop according to the adenoma–adenocarcinoma sequence [140]. Most sporadic CRCs lose APC function during adenoma development; then, mutations in other tumor suppressor genes or oncogenes, including *TP53*, *KRAS*, and *SMAD4*, lead to CRC initiation, promotion, and progression [141,142]. *APC* loss induces tumor-elicited inflammation, which leads to the disruption of the intestinal epithelial barrier, activation of IL-23-synthesizing myeloid cells, and expansion of tumor-resident IL-17-producing T lymphocytes, thereby stimulating proliferation of early tumor progenitors and causing adenoma growth [36,39]. By contrast, chronic inflammation caused by IBD, specifically ulcerative colitis or Crohn’s disease, can cause CRC and is termed colitis-associated cancer (CAC). Chronic inflammation and mucosal repair stimulate ROS and RNI production in the microenvironment, which induces mutations in the DNA and triggers tumor initiation [143]. CACs are characterized by the activation and recruitment of immune cells that produce pro-inflammatory cytokines, such as TNF, IL-6, IL-17, and IL-23, which lead to the propagation of an inflammatory and premalignant environment [144]. During IBD development, mutations in *IL12B*, *IL2, IFNG*, and *IL10*, which induce immune activation, and *XBP1, SLC9A4, SLC22A5*, and *SCL11A1*, which regulate ER stress, are observed [145,146]. Both sporadic CRC and CAC are relevant for inflammation; however, the order and etiology are different. In sporadic CRC, inflammation follows tumorigenesis; in CAC, inflammation precedes tumorigenesis [144]. Furthermore, although sporadic CRC and CAC entail similar mutations, the timing is different. In CAC, early detection of the *TP53* mutation and late detection of *APC* mutations are recognized, contrary to sporadic CRC [147].

MSI or deficiency of the MMR system occurs in approximately 15% of CRCs [148]. Microsatellites are DNA sequences distributed throughout the genome with repetitive structures, which are prone to replication errors in the case of dysfunction or mutation of the MMR system, referred to as deficient MMR (dMMR). Malfunction of the MMR system is highlighted by the accumulation of replication errors in the sequence of the microsatellites called MSI [149]. The MMR system consists of four major proteins, namely MLH1, MSH2, MSH6, and PMS2, which identify and correct DNA mismatches in microsatellites [150]. In dMMR-MSI-High (MSI-H) tumors, a higher expression of cytotoxic, Th1, Th2, and CD8^+^ T cell markers as well as macrophages and B cells was reported [151]. dMMR-MSI-H tumors demonstrate a higher mutational burden, which correlates with a higher expression of neoantigens, thus recruiting more cytotoxic CD8^+^ T cells [151]. T cell infiltration is a good predictive marker in patients with CRC, and dMMR-MSI-H tumors respond well to ICIs [152].

### 6.4. Liver Cancer

HCC occupies approximately 70% of liver cancers, whereas the second most frequent tumor type is intrahepatic cholangiocarcinoma (ICC) among various types of liver cancers [153]. The main risk factors associated with HCC are established, including hepatitis virus infection (HBV or HCV), alcohol consumption, and nonalcoholic fatty liver disease (NAFLD) [154]. Although the detailed mechanisms remain unclear, the incidence of NASH-related HCC, which is closely associated with obesity and type 2 diabetes, is increasing worldwide, especially in developed countries [155]. Although the cause of most ICCs is unclear, several risk factors, such as chronic biliary inflammation caused by primary sclerosing cholangitis, hepato-choledocholithiasis, and liver flukes, have been identified [156]. Notably, chronic inflammation is known to be involved in 80–90% of both types of liver carcinogenesis [154].

Chronic inflammation contributes to liver carcinogenesis through a cycle of cell death and regeneration, leading to the production of cell survival and proliferation signals that promote the formation of regenerative nodules, dysplasia, and cancer [85,157]. Carcinogenesis is associated with persistent cytokine production, which stimulates many liver cell types with various unique as well as redundant interactions. A predominant role of IL-4, IL-5, IL-8, and IL-10, which are Th2-like cytokines, in the microenvironment has been associated with a more aggressive and metastatic HCC phenotype compared to Th1-like cytokines [158].

Although inflammation releases many growth factors and cytokines, TNF has a central role in activating NF-κB and protecting transformed hepatocytes against apoptosis [95,159,160,161]. Chronic hepatitis causes immune cells to produce TNF; the released TNF then acts on TNF receptors in hepatocytes, activating IKK, which in turn activates NF-κB signaling and drives the expression of proliferation-promoting genes, such as *CCND1*; anti-apoptotic genes, such as *BCL2L1*; and the gene encoding TNF [159]. However, as per a previous study using a chemically induced mouse HCC model, hepatocyte-specific deletion of IKKβ increases HCC formation [85]. IL-6 is also a critical mediator of HCC development. IL-6 secreted by immune cells, such as macrophages and Kupffer cells, can activate the JAK–STAT signaling pathway and promote cell proliferation and survival [157,162]. IL-6 expression is also high in human ICC and promotes cell survival in a STAT3-dependent manner; moreover, a gene expression signature associated with IL-6–STAT3 signaling has been observed in a subset of human ICC [163,164]. IL-22, a cytokine capable of regenerating hepatocytes, acts through STAT3 and controls the activity of various cell survival and proliferation-associated genes; thus, inhibition of IL-22 is effective in suppressing HCC [165].

HBV contributes to HCC development directly as well as indirectly through hepatitis. The covalently closed circular DNA of HBV (*HBV cccDNA*) integrates with the host genome, which occurs during the early steps of clonal tumor expansion and induces both genomic instability and direct insertional mutagenesis of diverse cancer-related genes [166]. The HBx regulatory protein is required for viral replication, and is thought to contribute to HBV oncogenicity [167]. In addition to being required for viral replication, HBx contributes to HCC development by activating transcription factors through Ras–Raf–MAPK and NF-κB pathways, recruiting chromatin-modifying factors such as HDAC1 complex, and inducing genomic instability [168]. The landscape of genetic alterations in HBV-related HCC is also different from other types of HCCs, indicating a high rate of chromosomal alterations, *TP53* inactivation, and overexpression of fetal liver progenitor cell genes [169]. Chronic HCV infection promotes HCC development via direct virus-induced cellular programming, indirect host-related inflammatory response, and an overlapping host metabolic bystander effect [170,171].

Both immunogenic and tolerogenic immune responses occur in the immune microenvironment of HCC [172]. HCCs promote immunologic tolerance through the secretion of IL-10 and TGF-β. Tumor cells and TILs express immunosuppressive regulators, such as PD-1, PD-L1, and CTLA4, thereby allowing tumor growth and progression [173,174]. Efficacy of immunotherapy has been reported [175], but further correlative studies are necessary to understand the liver TME and establish successful anti-cancer immunotherapy.

### 6.5. Pancreatic Cancer

Exocrine tumors start in exocrine cells, where enzymes that help digest food are produced. The most common type of pancreatic cancer, namely pancreatic ductal adenocarcinoma (PDAC), is an exocrine tumor. Neuroendocrine tumors and other pancreatic neoplasms account for less than 5% of the remaining pancreatic cancers [176]. Microscopic precursors include pancreatic intraepithelial neoplasia (PanIN) and possibly atypical flat lesions. PanIN is frequently found in resected specimens of pancreas but is more common in specimens from patients with pancreatic cancer; PDAC is believed to arise from a spectrum of preneoplastic mucinous lesions like PanIN [177]. PanIN lesions are considered to progress stepwise to PDAC by accumulating multiple genetic alterations. Mutations in *KRAS* are considered to be the first stage of genetic alteration in PanIN [178]. Although *KRAS* mutations are frequently found in low-grade PanIN and high-grade PanIN, mutations in *CKN2A*, *TP53,* and *SMAD4* are usually only found in high-grade PanIN and more often in invasive PDAC [179]. Chronic pancreatitis induces the *KRAS* activating mutation, which is followed by mutations in tumor suppressor genes *CDKN2A* (p16), *TP53,* and *SMAD4* [180].

Autophagy induced by the pancreatitis-associated protein, vacuole membrane protein 1 (VMP1), cooperates with Kras^G12D^ to promote PDAC initiation [181]. Kras^G12D^ also induces IL-1α expression through AP-1 activation, which leads to NF-κB activation in PDAC cells [182]. Activation of NF-κB drives various signaling pathways, such as Notch, EGFR, and PI3K [183]. NF-κB can be activated during chronic pancreatitis by enhanced inflammatory cells and macrophages, subsequently elevating the level of cytokines and chemokines, which correlates with enhanced NF-κB signaling [95,184].

Chronic inflammation can lead to the production of pro-inflammatory cytokines, such as TNF and IL-6. IL-6 activates intrinsic molecular pathways, including JAK–STAT3, MAPK, and PI3K signaling pathways. The IL-6–STAT3 axis can promote pancreatic cancer cell growth and progression [185]. This axis can be inhibited by SOCS3 (suppressor of cytokine signaling 3); however, SOCS3 activity is often suppressed in cancer cells [186]. A recent study has revealed that LIF, one of the IL-6 family cytokines, produced from pancreatic stellate cells, also promotes pancreatic tumorigenesis and is an attractive therapeutic target and circulating marker [187]. TNF, produced by both myeloid cells and pancreatic cancer cells, stimulates the production of other cytokines and chemokines and enhances primary tumor growth, metastases, angiogenesis, and chemoresistance [188]. TNF is also related to the establishment of the immunosuppressive TME [188].

To date, studies exploring therapies targeting *KRAS, BRAF, BRCA1/2*, *ATM,* and *CDKN2A* mutations have not shown meaningful benefit in PDAC. ICIs have not been shown as promising reagents of PDAC, even though PD-L1 expression has been confirmed in a subset of patient samples [189]. On the other hand, the dual blockade of IL-6 and PD-L1 reduced tumor progression in an experimental model [190]; therefore, a combination of ICIs and other agents can be a promising therapeutic strategy.

The role of inflammation in GI cancers is summarized in Table 1.

### 6.6. Lung Cancer

Lung cancer, the cancer which has killed the American people the most since the 1950s, remains the biggest cause of death among all cancers today (American Cancer Society 2020 Cancer Facts & Figures). Despite recent therapeutic advances, such as ICIs and molecular targeting therapies against specific oncogenic mutations, the prognosis of lung cancer remains as low as 19% in 5-year relative survival rates in the U.S. The correlation between global tobacco consumption and the increase in smoking-attributable lung cancer deaths has been established [207], and the global incidence is estimated to increase in the first half of this century. As tobacco causes approximately 80% of lung cancer cases and chronic obstructive pulmonary disease is known to be linked to the development of lung cancer independently of smoking dosage [208], the role of inflammation in the progression of lung cancer has long been recognized [209].

The development of cutting-edge techniques has advanced lung cancer research from the perspective of cancer immunology. Major components of the lung cancer microenvironment include CAFs around alveoli, ECM around airway epithelium, and infiltrating immune cells, such as lymphocytes, TAMs, and MDSCs [210,211].

CAFs are activated by cytokines, such as TGF-β, PDGF, and FGF-2, which are secreted by lung cancer cells [212]. CAFs produce collagen and remodel tissue structure by enhancing tissue stiffness. CAFs are functionally altered through metabolic regulation and autophagy, and this may be linked to increased collagen production [213]. This remodeling in turn secretes TGF-β and positively feeds back to the immunosuppression within the TME [214]. CAFs can also alter the characteristics of lung cancer cells by interacting through ROS, TGF-β [215], and IGF-2/IGF-1R/Nanog paracrine signaling [216]. Interestingly, immune cell function can be regulated by CAFs. For example, CAFs contribute to the induction of Tregs via PGE_2_ production [217] and the inhibition of CD8^+^ T cell activation through the expression of PD-L1, PD-L2, and Fas ligand [218].

ECM is categorized into collagens, glycoproteins, and proteoglycans/glycosaminoglycans. Each of these comprises multiple components, which associate with lung cancer progression and metastasis through multiple mechanisms [212,219]. Related to the known increased risk of lung cancer in patients with idiopathic pulmonary fibrosis, the constant mechanical stress in the pulmonary ECM during respiration can produce more TGF-β [220], generating an immune-suppressive milieu.

There are many reports on immune cell profiling in lung cancer [210]. Unfortunately, widely-used immune parameters, such as Treg:CD8 ratio [221] and MDSC counts, have not been shown as promising prognostic markers for lung cancer. Possible explanations for this include: (1) more detailed analysis with a larger sample cohort is warranted, or (2) immune profiling should be based on the pattern of genetic mutations in each patient with lung cancer. Accumulating evidence suggests that the gene mutation patterns in lung cancers have a significant impact on their immunological properties. A notable example is the inactivation of *liver kinase B1* (*LKB1*), a tumor suppressor, which reportedly promotes neutrophil recruitment and pro-inflammatory cytokine (IL-1β, IL-6, IL-33, and CXCL7) production to suppress T cell infiltration and function [222]. Moreover, mutations in *KRAS*, a major driver mutation of non-small cell lung cancer (NSCLC), are correlated with elevated pro-inflammatory conditions, which favor the immune-suppressive microenvironment [32,223]. Furthermore, a recent study suggested that immune landscape of the *KRAS*-mutant lung cancer is greatly affected by the co-occurrence of mutations of *LKB1, MYC, KEAP1, or TP53* genes [224]. Importantly, the genetic mutation-induced alteration of the TME is also notable in its different responsiveness to cancer immunotherapy.

Finally, we discuss two key regulators of inflammation, NF-κB and COX-2. NF-κB signaling plays a critical role in the pathogenesis of lung cancer as well as in other cancer types [95,225]. For example, tobacco smoke reportedly promotes lung tumorigenesis by triggering IKKβ- and JNK1-dependent inflammation [17]. According to a recent report, smoking-induced chronic inflammation activates lymphotoxin β-receptor (LTβR) and its downstream noncanonical NF-κB signaling in lung epithelial cells, resulting in airway fibrosis [226]. Notably, NF-κB contributes to the suppression of anti-tumor immunity by inducing PD-L1 expression in cancer cells. For example, the NSCLCs with mutated *EGFR* express a higher level of PD-L1 than those with wild-type EGFR, and this phenomenon can be inhibited by the EGFR- tyrosine kinase inhibitor gefitinib in an NF-κB-dependent manner [227,228].

COX-2 and its enzymatic product PGE_2_ play critical roles in the formation of the lung-cancer promoting immune microenvironment. For example, PGE_2_ promotes Foxp3^+^ Treg activation [217]. In addition, a previous report demonstrated that COX-2–PGE_2_ drives cancer-promoting inflammation, while suppressing anti-tumor immunity. Specifically, tumor-derived PGE_2_ directly induces the production of cancer-promoting inflammatory mediators, such as IL-6, CXCL1, and G-CSF, by myeloid cells, while preventing the activation of anti-tumor type I immune responses [229]. This report also suggests the synergistic effect of inhibition of COX and anti-PD-1 blockade. Whether inhibitors of the COX-2–PGE_2_ axis, such as aspirin, can actually reduce the risk of lung cancer has been a controversial issue [230]. However, interestingly, a recent report suggested that aspirin can block the formation of metastatic intravascular niches in the lung by inhibiting platelet-derived COX-1/thromboxane A2 (TXA2) [231].

### 6.7. Prostate Cancer

Prostate cancer (PCa) has been a leading cause of mortality in men in the U.S. [232]. Among several putative risk factors involved in the development of PCa, such as mutations [233] in genes associated with DNA repair and androgen receptor (AR) activation [234,235,236], elevated chronic inflammation in the prostatic microenvironment is known to be correlated with cancer development [237,238]. This is also corroborated by a clinical prospective study [239], but the molecular mechanisms of how inflammation clinically affects PCa development have only begun to be clarified [240].

Here, we discuss and review the recent findings in this field. Inflammation as a cause of carcinogenesis in PCa can be separately understood by cancer cell-extrinsic and -intrinsic mechanisms. As for external factors, microbiome and fatty diet or obesity are the most notable causes of PCa.

The microbiome, which impacts carcinogenic inflammation, is a component of the urinary and gastrointestinal system. Innovative technical advances in NGS have revealed characteristics of microbial flora in the gut and prostate. A recent prospective pilot study showed a higher abundance of the specific bacteria in the gut of patients with PCa compared to controls [241]. Another group evaluated viral, bacterial, and fungal load in resected samples of PCa and found that viral and Helicobacter integrations are predicted to affect the expression of several cellular genes associated with oncogenesis [242]. Another plausible mechanism is that *Cutibacterium acnes* induces macrophagic inflammation, including type I IFN, through the STING pathway [243]. Moreover, related to therapy, some gut microbiota can associate with the clinical response to AR axis-targeted therapies [244] or anti-PD-1 immunotherapy [245]. The detailed molecular mechanisms on how these microbiotas induce PCa-promoting chronic inflammation remain to be elucidated, so further research is warranted.

A HFD or obesity can contribute to PCa carcinogenesis [246,247], while a prudent dietary pattern can reduce PCa risk. This observation has been corroborated with epidemiological studies in Asian countries, whose PCa incremental incidences follow those in Western countries [248]. There are multiple mechanisms of carcinogenesis induced by obesity, and most of them lead to the increase in adipocyte number. These cells produce pro-inflammatory adipokines, such as IL-6 [249] and CCL7 [250] which favor cancer progression. Moreover, *MYC*, an oncogenic driver in PCa [251,252], can be amplified by HFD-induced metabolome rewiring [253]. In addition, chemical injury caused by uric acid, and physical trauma caused by corpora amylacea [240], also act as external factors that trigger PCa-promoting chronic inflammation. Another proposed mechanism includes TLR4 activation by some kinds of irritants, inducing NF-κB and promoting PCa progression [254,255,256].

An example of a cancer cell-intrinsic mechanism is the mutations of oncogenes involved in the important signaling pathways in PCa, which affect the pro-inflammatory TME [237]. c-Myc-driven PCa favors an immunosuppressive TME by producing cytokines, such as CXCL2, CCL5, and TGF-β1 [257]. Alterations in the WNT signaling pathway reportedly occur in around 20% of PCa cases [258], and WNT-β-catenin activation correlates with immunosuppression within the TME, represented by a lower CD8^+^/FOXP3^+^ ratio [259]. Loss of *PTEN* is observed in as many as 40% of PCa cases [258], and this mutation promotes PCa progression by activating CXCL8 [260] or NF-κB signaling and increasing the infiltration of MDSCs, Treg, and M2 macrophages [261]. Interestingly, *PTEN*-null tumor cells exhibit a prominent senescent phenotype [262], and are characterized by the excessive production of several tumor-promoting inflammatory cytokines, the so-called SASP, and the establishment of an immunosuppressive TME by themselves [263].

The interaction between PCa and immune cells in the TME, specifically TAMs, MDSCs, and mesenchymal stem cells (MSCs), is noteworthy in terms of inflammation-induced PCa progression. According to recent reports, PCa induces the functional polarization of TAMs toward a tumor-promoting phenotype through CXCL2–CXCR2 signaling, and this axis can be a promising therapeutic target [264,265]. MDSCs are immunosuppressive cells in the TMEs of multiple cancers, including PCa [266]. Notably, MDSCs confer castration resistance in PCa; IL-23 secreted by MDSCs activates AR signaling in PCa, promoting cell survival and proliferation in androgen-deprived conditions [235]. In addition, MSCs suppress T cell proliferation in the TME by upregulating PD-L1 and PD-L2, thereby promoting tumor progression [267].

Attempts to introduce immunotherapy into the treatment of PCa are currently underway. In particular, the combination of AR-targeting agents and ICI*s* as a promising therapeutic strategy has been attracting attention [268]. However, there are concerns that AR-directed therapies, when combined with ICIs, may induce cross-resistance because the production of immune checkpoint molecules, such as PD-L1, is likely regulated downstream of AR [269]. Therefore, further research is needed for the clinical application of the combination therapy in the future.

### 6.8. Breast Cancer

The relationship between inflammation and breast cancer has been extensively investigated. Here, we discuss the recent advances in this field, especially by focusing on the pathology of inflammatory breast cancer (IBC), and the role of obesity-associated inflammation in breast cancer.

IBC is a rare subtype of breast cancer, comprising only 3% of all breast cancer cases. However, it is known to be aggressive and accounts for 7–10% of all breast cancer mortalities. Considering that IBC patients tend to be younger than non-IBC patients, the clarification of underlying mechanisms and development of targeted therapies for IBC are sorely warranted [270]. IBC, with a distinct phenotype from non-IBC, is characterized by several clinical features such as dermal–lymphatic invasion and intralymphatic tumor emboli [271]. In addition, although ALDH1 [272] and E-cadherin [273] have been identified as indicators of poor prognosis, the genomic characterization-based classification of IBC and non-IBC has not yet succeeded [270]. Whereas distinguishable markers have not yet been identified, several inflammatory pathways have been reported to be characteristic of IBC. Elevation of cytokines such as IL-6 [274] and IFNα [275] are reportedly notable in the pathogenesis of IBC. Blocking of IL-6 by neutralizing antibody or statin treatment can suppress tumor progression, suggesting alteration of the TME. In addition to the JAK–STAT signaling pathway, EGFR and COX-2 pathways contribute to IBC progression. Anti-EGFR antibody therapy has shown promise, and its phase II clinical trials are ongoing [276]. Moreover, JAK2–STAT3 hyperactivation can be a mechanism of chemotherapy resistance in IBC [277]. This pathway may also play a role in the survival of cancer stem-like cells in breast cancer [278,279]. COX-2-driven inflammation has been reported to promote the progression of several types of cancers [229], including IBC. Chronic inflammation in IBC can enhance EGFR and COX-2 signaling, which may act in conjunction to maintain cancer cell stemness [280]. Additionally, immune cell profiling is essential for understanding the pathology of IBC [270]; although several types of immune cells, including TAMs [281], DCs, T cells, and MSCs [274], have been suggested to play important roles in IBC, further robust evidence is needed to accept them as appropriate therapeutic targets.

The interplay between adipocytes and breast cancer cells is critical for disease progression and chemoresistance [282]. These processes are associated with chronic inflammation induced by white adipose tissue (WAT) adipocytes, which are abundant in obese patients [283]. WAT releases free fatty acids, which activate TLR4, resulting in the secretion of pro-inflammatory cytokines such as TNF and IL-1β via NF-κB activation [284]. Moreover, adipocytes deregulate COX-2 and sex hormones, enhancing enzymatic conversion of androstenedione to estrone, leading to elevated aromatase activity [285,286]. Furthermore, adipocytes promote aromatase activation via hyperinsulinemia or via stimulating PGE_2_ secretion from malignant epithelial cells [287].

Among several investigated adipokines that are associated with breast cancer, one of the most notable is leptin. Binding of overexpressed leptin to leptin receptors (LEPRs) in peritumoral fat and breast cancer cells stimulates multiple signaling events, such as JAK2–STAT3, MAPK–ERK, and PI3K–AKT–mTOR pathways, thereby enhancing tumor proliferation and survival [285,288]. These cytosolic signaling events can activate NF-κB and HIF-1α, leading to VEGF-A activation [289]. mTOR signaling activates the translation of pro-inflammatory cytokines such as TNF IL-1, and IL-6 [288]. Leptin–LEPR signaling also cooperatively functions with other multiple signaling pathways, such as EGFR, Notch, IL-1, and the estrogen receptor, to enhance the proliferation, migration, invasion [290], and self-renewal activity of breast cancer stem cells [291]. Recent robust data demonstrating the role of leptin, IGF-1, adipocytokines, and obesity-related hormones in the pathology of breast cancer has clarified the possibility of disease prevention through lifestyle alteration and specific signaling-targeted inhibition [287,292].

Furthermore, obesity and adipocytes are associated with resistance to cancer chemotherapy [293], including in breast cancer [294]. The alteration of lipid metabolism in the TME is one of the important mechanisms of obesity-related chemoresistance. Fatty acid oxidation regulated by leptin–LEPR signaling has been shown to be related to chemoresistance [295]. Moreover, breast cancer is characterized by increased HIF production [296], which in turn promotes IL-6 production in the adipocytes and myeloid cells in obese patients, thereby promoting resistance to anti-VEGF-A therapy [297]. Inflammasome activation is one form of the tumor-promoting inflammatory responses, and recent studies have demonstrated that inflammasomes also participate in the pathogenesis of breast cancer [298]. TME in the context of obesity increases tumor-infiltrating myeloid cells with activated NLRC4 inflammasomes, thereby activating IL-1β and driving disease progression through adipocyte-mediated *VEGFA* expression and angiogenesis [299]. Obesity also accelerates tumor progression or metastasis by altering the immune cell landscape [300]. An intriguing report showed that obesity led to the CXCR2-mediated accumulation of FasL^+^ granulocytic MDSCs, resulting in increased apoptosis of tumor-infiltrating CD8^+^ T cells and immunotherapy resistance [301].

Even in the absence of obesity-associated inflammation, the immune microenvironment greatly affects the pathogenesis of breast cancer. The expression of CCL2 in breast cancer in conjunction with the infiltration of CCR2-expressing inflammatory macrophages is correlated with poor prognosis and metastatic human breast cancer [302]. Though blocking this CCL2–CCR2 signaling axis seems to be a promising therapy, its cessation may cause recurrence of the disease in an IL-6- and VEGF-A-dependent manner [303]. Another intriguing study has demonstrated that the crosstalk between T cells and neutrophils promotes metastatic breast cancer [49]; tumor-derived IL-1β elicits IL-17-producing γδ T cells, which drives G-CSF-dependent expansion and the functional polarization of neutrophils toward a CD8^+^ T cell-suppressive phenotype, thereby promoting subsequent metastasis formation in distal organs.

Collectively, accumulating evidence demonstrates the association of inflammation with breast cancer initiation, progression, and metastasis. Nevertheless, the role of inflammation in breast cancer is still complicated. For example, blockade of WNT secretion in *TP53*-null breast cancer cells reportedly suppresses IL-1β production by macrophages and subsequent neutrophil infiltration, resulting in reduced metastasis [33]. On the contrary, another group has reported that high IL-1β expression in breast cancer is associated with better overall survival and distant metastasis-free survival, preventing metastasis-initiating cells from generating highly proliferative E-cadherin-positive cells [304]. In summary, extensive investigation is required for the development and future clinical application of inflammation-targeted breast cancer therapies [305].

### 6.9. Hematological Malignancies

Hematological malignancies result from the clonal expansion of hematopoietic stem cells (HSCs) with cytogenetic abnormalities. Signals provided by the surrounding environment (niche) tightly regulate the self-renewal and differentiation of HSCs to maintain their quiescence state. However, this characteristic quiescence may be a double-edged sword, which leads to the acquisition of genomic rearrangements owing to their reliance on nonhomologous end joining-mediated DNA repair [306]. Recent advances in molecular biology, especially large-scale whole-exome sequencing studies, have enabled clarification of how a premalignant state progresses to leukemia [307].

Clonal hematopoiesis of indeterminate potential (CHIP) is a clinical entity that presents clonal mutation of genes associated with malignancy in humans, especially those involving epigenetic regulation, such as *DNMT3A*, *TET2*, and *ASXL1* [308,309,310] or spliceosomes, such as *SF3B1*, *SRSF2*, and *U2AF1*. These mutations have been extensively reported to be common drivers in myeloid malignancies, such as acute myeloid leukemia (AML), myelodysplastic syndrome (MDS), and myeloproliferative neoplasm (MPN), and mutation acquisition occurs not only at the precancerous state but also after chemotherapy [311,312]. Moreover, CHIP was reported to be associated with adverse prognosis of both myeloid and lymphoid malignancies.

Recent studies have investigated the mechanism that potentiates the progression of pre-leukemic disorders, such as MDS and MPN, and how the selective propagation of malignant clones or apoptosis resistance is regulated. These processes are closely related to genetic mutation patterns and have been discussed from a multi-factorial point of view [313,314]. For example, mutations in the genes associated with cell cycle regulation and DNA damage response, such as *TP53* and *PPM1D*, or cellular growth signaling, such as *JAK2*, confer resistance to environmental stress and enhance survival advantages [315]. In addition, two major DNA methylation regulator genes, *DNMT3A* and *TET2,* are often mutated in hematological disorders, despite possessing the opposite epigenetic functions. TET2 is an enzyme involved in DNA demethylation and DNMT3A in DNA methylation. In some AML cases, the *FLT3^ITD^* mutation cooperates with the *TET2* mutation to potentiate leukemogenesis [316].

Several epidemiologic reports have suggested a correlation between hematological malignancies and inflammation, such as infection or autoimmune diseases [317,318]. Some immune-related genes were reportedly upregulated in hematopoietic stem and progenitor cells (HSPCs) in patients with MDS [319,320,321]. Recent reports have demonstrated that elevated innate immune signaling impacts the progression of hematological malignancies. For example, MDS HSPCs manifest NLRP3 inflammasome activation [322] and *Tet2*-deficient hematopoietic stem and progenitor cells exhibit hyperactivation of the IL-6–STAT3 pathway [323]. Another report suggests that *TET2* mutation can lead to elevated IL-6 production because TET2 generally suppresses IL-6 expression through the recruitment of HDAC2 to the *IL6* promoter [324,325].

Interestingly, the mechanistic importance of NF-κB in hematological malignancies, such as AML, MDS, and multiple myeloma, was recently reported [95,326]. A recent study reported that *U2AF1*, a splicing factor often mutated in MDS, induces the expression of the gene encoding the oncogenic isoform of IRAK*4* (IRAK4-L), thereby activating NF-κB signaling [319]. The same group subsequently reported that an adaptive response to inflammation by MDS HSPCs enhances switching from canonical to noncanonical NF-κB signaling, depending on TLR–TRAF6-mediated activation, and the inhibition of noncanonical NF-κB signaling could prevent the progression of MDS [327].

In addition to cell-intrinsic mechanisms, alterations in the microenvironment in the bone marrow (BM) have been reported to contribute to MDS progression and hematopoietic failure [328,329]. This HSPC-extrinsic mechanism is supposed to be regulated by immune cells [307], mesenchymal stroma [330], and cytokines [331]. According to a recent report, endogenous DAMP molecules S100A8/9, secreted from p53-activated mesenchymal niche cells, drive genotoxic stress in HSPCs, and contribute to the disease evolution of human pre-leukemia through TLR4 receptor signaling [332]. S100A9 also engages in the pathogenesis of MDS by inducing the activation and expansion of MDSCs in the BM through the S100A9–CD33 signaling pathway [333].

Intriguingly, CHIP itself increases inflammation and can cause nonmalignant diseases. CHIP has been reported to be a risk factor for developing coronary heart disease (CHD) in humans and accelerated atherosclerosis in mice [334]. Jaiswal et al. reported that carriers of CHIP have a CHD risk as much as 1.9 times greater compared to noncarriers, and are characterized by excessive chemokine production in macrophages due to *TET2* deficiency. This finding is consistent with another report that *TET2* mutation in blood cells enhances atherosclerosis through the elevation of NLRP3 inflammasome-mediated IL-1β secretion [335].

Importantly, the age-related increase in somatic mutations can provide a growth advantage, called age-related clonal hematopoiesis. HSC is also known to exhibit age-related myeloid-biased differentiation. This process can be regulated by cell-extrinsic inflammatory stimulus, such as through TLRs and type I IFNs signaling. [336]. How this age-related lineage bias is enhanced by elevated inflammation and finally contributes to the progression of myeloid malignancies through clonal selection remains to be elucidated [337].

Clinical trials of inflammation-targeted therapies are underway for patients with MDS [338], including TLR signaling inhibition [339,340], MDSC elimination [341], NLRP3 inflammasome inhibition [342], and IL-1β inhibition [343]. These are in preliminary stages because how innate immunity and inflammation impacts hematological oncogenesis has not been fully understood yet. However, insights into this field will clarify the previously unknown mechanism of refractory malignancies and lead to the development of better therapeutic strategies [344].

The role of inflammation in lung, prostate, and breast cancers and hematological malignancies is summarized in Table 2.

## 7. Conclusions and Perspective

Katsusaburo Yamagiwa artificially induced cancer in experiments more than 100 years ago. Subsequent studies have revealed the molecular mechanisms through which inflammation promotes tumorigenesis and metastasis. As mentioned above, some of the mechanisms are common regardless of cancer type, whereas others are specific to cancer types. There is growing evidence that chronic inflammation and pro-inflammatory signaling pathways are attractive targets for preventing and treating cancer. Specifically, pro-inflammatory cytokines, such as TNF and IL-6, inflammation-related kinases, such as IKKβ and JAK, and inflammation-related transcriptional factors, such as NF-κB and STAT3, are potential therapeutic candidates in cancer. However, suppression of inflammation and inflammation-related molecules for a long period results in immunosuppression due to their physiological roles, which increases the risk of severe infection. Moreover, suppression of inflammation might inhibit tissue repair and regeneration and anti-tumor immunity. Therefore, identifying key molecules, which are specific for cancer-related inflammation and certain types of cancer, and targeting such molecules to avoid side effects, is extremely important. Further studies on inflammation and cancer are still necessary to achieve that goal.

## Figures and Tables

**Table 1:** Role of inflammation in GI cancers.

Types of Cancers	Features	References
Esophageal cancer	IL-6 and IL-8 are produced by ESCC cells and they are implicated in immune evasion via STAT3 activation.	[110]
	LIF produced by ESCC cells is necessary for tumor cell proliferation and migration/invasion.	[103]
	IL-6 induces STAT3 and ERK1/2 signaling and IL-6 knockout attenuates ESCC cell proliferation.	[191]
	IL-6 secreted by CAFs induces EMT and therapy resistance in esophageal adenocarcinoma.	[192]
	*miR-204-5p* functions as a tumor suppressor by directly inhibiting IL-11 expression.	[193]
Gastric cancer	IL-8 and IL-17 are induced by the activation of NF-κB pathway and initiate gastric neoplasia.	[122]
	CD8+ T cell infiltration is enhanced in EBV+ GC.	[129]
	CAFs that secret IL-6 enhances cancer cell migration and EMT by activating STAT3.	[132]
	IL-11-producing CAFs facilitate the chemotherapeutic drugs resistance of GC cells.	[133]
	IL-22 enhances the viability of GC cells through upregulating the JNK signaling pathway.	[134]
	IL-22 stimulation promotes the migration and invasion of GC cells by regulating the AKT/MMP-9 signaling axis.	[135]
	IL-22-expressing CAFs promote GC cell invasion via STAT3 and ERK signaling.	[136]
Colorectal cancer	APC loss activates IL-23-synthesizing myeloid cells and expands tumor-resident IL-17-producing T lymphocytes.	[36]
	Th1, Th2, CD8+ T cells, macrophages, and B cells are recruited in MSI-H tumors compared to microsatellite stable (MSS) cancers.	[151]
	IL-6 promotes early CAC tumorigenesis via STAT3 activation.	[194]
	Inhibition of IL-11 signaling attenuates colitis-promoted carcinogenesis.	[195,196]
	TGF-β signaling in CD4+ T cells promotes the emergence of IL-22-producing Th17 cells and thereby colorectal tumorigenesis.	[197]
	Excessive IL-22 in the colon cancer microenvironment leads to tumor growth, inhibition of apoptosis, and promotion of metastasis via STAT3 activation.	[198]
Liver cancer	Th2-like cytokines such as IL-4, IL-5, IL-8, and IL-10 are associated with a more aggressive and metastatic HCC phenotype.	[158]
	IL-6 secreted by immune cells such as macrophages and Kupffer cells activates inflammatory signaling pathways in hepatocytes via JAK–STAT3 and NF-κB and promotes cell proliferation.	[157]
	IL-22 controls the activity of a wide variety of HCC cell survival and proliferation genes.	[165]
	HCCs promote immunologic tolerance through the secretion of IL-10 and TGF-β.	[173]
	Inhibition of IL-6 signaling turns macrophages into M1-type and reduces HCC tumor formation.	[199]
	Direct suppression of IKKβ by *miR-451* inhibits HCC cell proliferation.	[200]
Pancreatic cancer	Chronic inflammation leads to production of pro-inflammatory cytokines such as TNF and IL-6. IL-6 activates JAK–STAT3 signaling and promotes pancreatic cancer cell growth and progression.	[185]
	LIF produced by pancreatic stellate cells promote pancreatic tumorigenesis.	[187]
	TNF produced by myeloid cells and pancreatic cancer cells stimulates the production of other cytokines which enhances primary tumor growth.	[188]
	Persistent STAT3 activation mediated by loss of p53 promotes pancreatic tumor growth.	[201]
	Tumor-induced IL-6 results in the change of metabolic response and thus impairs anti-tumor immunity.	[202]
	IL-6 stimulates pancreatic cancer cell proliferation and survival.	[203]
	LIF expression is induced by oncogenic *KRAS* in PDAC and LIF depletion prevents engraftment in pancreatic xenograft models.	[204]
	ILC3s promote the proliferation, migration, and invasion of pancreatic cancer cells by secreting IL-22 to activate AKT signaling.	[205]
	IL-22 promotes acinar to ductal metaplasia, stem cell features, and increased expression of EMT markers.	[206]

**Table 2:** Role of inflammation in lung, prostate, and breast cancers and hematological malignancies.

Types of Cancers	Features	References
Lung cancer	Stage I lung adenocarcinoma lesions already harbor significantly altered T cell and NK cell compartments.	[211]
	CAFs and matched normal fibroblasts show 46 differentially expressed genes, encoding significantly enriched extracellular proteins regulated by the TGF-β signaling pathway.	[214]
	CAFs support T cell suppression within the tumor microenvironment by a mechanism dependent on immune checkpoint activation.	[218]
	CAFs constitute a supporting niche for cancer stemness through IGF-2/IGF-1R signaling and this blockade inhibits Nanog expression.	[216]
	Stabilized HIF-1α protein expression inhibits the TGF-β-stimulated appearance of EMT phenotypes across cell types and species.	[219]
	Co-occurring genomic alterations, particularly in *TP53* and *LKB1*, have emerged as core determinants of oncogene-driven lung cancer subgroup.	[224]
	Tumor-promoting inflammation and immune modulation caused by *KRAS* mutation leads to immune escape in the TME.	[32]
	IL-6 is a potential druggable target for prevention and treatment of *Kras*-mutant lung tumors.	[223]
	NF-κB can be a potential companion drug target, together with EGFR, in *EGFR*-mutant lung cancers.	[225]
	Repetitive exposure to tobacco smoke promotes tumor development through Kras activation in lung epithelial cells.	[17]
	Aspirin blocks formation of metastatic intravascular niches by inhibiting platelet-derived COX-1/TXA2.	[231]
Prostate cancer	Exposure to environmental estrogens increases the risk of PCa.	[234]
	IL-23 secreted by myeloid cells drives castration-resistant PCa.	[235]
	Inflammation and atrophy are involved in prostate carcinogenesis and the microbiome play an important role in establishing an inflammatory microenvironment.	[238]
	A HFD drives metastasis in a *PTEN*-null mouse model of PCa, and an SREBP signature was highly enriched in metastatic human CaP.	[247]
	A HFD fuels PCa progression by rewiring the metabolome and amplifying the *MYC* program.	[253]
	Senescence induced by *PTEN* deficiency or chemotherapy limits the progression of PCa and *TIMP1* deletion allows senescence to promote metastasis.	[262]
	Adipocytes from periprostatic adipose tissue support the directed migration of PCa cells through CCR3/CCR7 axis promoted by obesity.	[250]
	Disruptions of *CHD1* that define a subtype of ETS gene family fusion-negative PCa and *ETS2* are also deregulated through mutation.	[236]
	Activation of PI3K–AKT–mTOR and MAPK signaling pathways in prostate tumors cooperate to upregulate c-Myc.	[252]
	In metastatic castration-resistant PCa patients, aberrations of AR, ETS genes, *TP53*, and *PTEN* are frequent, with *TP53* and AR alterations enriched in mCRPC compared to primary PCa	[258]
	CAFs and M2-polarized macrophages synergize during prostate carcinoma progression.	[264]
	*PTEN*-null prostate tumors are infiltrated by TAMs expressing CXCR2, and activation of CXCR2–CXCL2 polarizes macrophages toward anti-inflammatory status.	[265]
Breast cancer	eIF4GI reprograms the protein synthetic machinery for increased translation that promotes IBC tumor cell survival and formation of tumor emboli.	[273]
	There is a crosstalk of immune and stromal cells in the local tumor microenvironment and IBC through IFNα signaling.	[275]
	The JAK2–STAT3 signaling pathway is required for growth of CD44+CD24- stem cell-like breast cancer cells in human tumors.	[279]
	Leptin signaling contributes to the metabolic features and shapes the tumor microenvironment.	[288]
	A fasting-mimicking diet promotes long-lasting tumor regression and reverts acquired resistance to drug treatment.	[292]
	JAK–STAT3-regulated fatty acid beta-oxidation is critical for breast cancer stem cell self-renewal and chemoresistance.	[295]
	BC patients with obesity harbored increased systemic concentrations of IL-6 and/or FGF-2 and their tumor vasculature was less sensitive to anti-VEGF-A treatment.	[297]
	Obesity induces an increase in tumor-infiltrating myeloid cells with an activated NLRC4 inflammasome in breast cancer.	[299]
	Inhibition of CCL2–CCR2 signaling blocks the recruitment of inflammatory monocytes and inhibits metastasis of breast tumors in a murine model.	[302]
	Inhibition of CCL2 and IL-6 markedly reduces metastases of breast cancer.	[303]
	Targeting breast cancer cell-initiated domino effect within the immune system (the γδ T cell/IL-17/neutrophil axis) inhibits metastasis.	[49]
	Blockade of WNT secretion in *TP53*-null cancer reverses macrophage production of IL-1β and subsequent neutrophilic inflammation, resulting in reduced metastasis.	[33]
	Ablation of the pro-inflammatory response or inhibition of the IL-1 receptor relieves the differentiation block of metastasis-initiating cancer cells into highly proliferative progeny, and results in metastatic colonization of breast cancer.	[304]
Hematological malignancies	Germline genetic variation can shape somatic variation in hematopoietic stem cells, leading to CHIP.	[308]
	Age-related clonal hematopoiesis is associated with increases in the risk of hematologic and cardiovascular disease.	[309]
	Preleukemic HSCs can survive induction chemotherapy, identifying these cells as a reservoir for the re-evolution of relapsed disease.	[311]
	Mutations in *PPM1D*, a DNA damage response regulator, drive clonal hematopoiesis in response to cytotoxic chemotherapy.	[312]
	*JAK2V617F* promotes clonal selection by conferring TNF resistance, while simultaneously generating a TNFα-rich environment.	[315]
	Chronic immune stimulation acts as a trigger for AML/MDS development.	[317]
	*U2AF1* mutations induce oncogenic IRAK4 isoforms and activate innate immune pathways in myeloid malignancies.	[319]
	NLRP3 inflammasome functions as a driver of the myelodysplastic syndrome phenotype.	[322]
	*TET2*-deficient mature myeloid cells and HSPCs increase in response to inflammation, resulting in production of inflammatory cytokines and resistance to apoptosis.	[323]
	Upon inflammation, MDS HSPCs switch from canonical to noncanonical NF-κB signaling, which is dependent on TLR–TRAF6-mediated activation of A20.	[327]
	Perturbation of specific mesenchymal stromal cells can disorder function and apoptosis of heterologous cells, and disrupt tissue homeostasis.	[329]
	Overproduction of niche factors such as CDH2, IGFBP2, VEGF-A, and LIF is associated with the ability of MSCs to enhance MDS expansion.	[330]
	Activation of p53-S100A8/9-TLR inflammatory signaling axis in the mesenchymal niche predicts leukemic evolution and progression in MDS.	[332]
	Leukemic stem cells isolated from *de novo* AML patients are uniquely reliant on amino acid metabolism for oxidative phosphorylation.	[344]

## Data Availability

No new data were created or analyzed in this study.
